# Activation and Speciation Mechanisms in Class A GPCRs

**DOI:** 10.1016/j.jmb.2022.167690

**Published:** 2022-06-18

**Authors:** Bentley Wingert, Pemra Doruker, Ivet Bahar

**Affiliations:** Department of Computational and Systems Biology, School of Medicine, University of Pittsburgh, Pittsburgh, PA 15213, USA

**Keywords:** G-protein coupled receptors, signature dynamics, conformational landscape, principal component analysis, elastic network model

## Abstract

Accurate development of allosteric modulators of GPCRs require a thorough assessment of their sequence, structure, and dynamics, toward gaining insights into their mechanisms of actions shared by family members, as well as dynamic features that distinguish subfamilies. Building on recent progress in the characterization of the signature dynamics of proteins, we analyzed here a dataset of 160 Class A GPCRs to determine their sequence similarities, structural landscape, and dynamic features across different species (human, bovine, mouse, squid, and rat), different activation states (active/inactive), and different subfamilies. The two dominant directions of variability across experimentally resolved structures, identified by principal component analysis of the dataset, shed light to cooperative mechanisms of activation, subfamily differentiation, and speciation of Class A GPCRs. The analysis reveals the functional significance of the conformational flexibilities of specific structural elements, including: the dominant role of the intracellular loop 3 (ICL3) together with the cytoplasmic ends of the adjoining helices TM5 and TM6 in enabling allosteric activation; the role of particular structural motifs at the extracellular loop 2 (ECL2) connecting TM4 and TM5 in binding ligands specific to different subfamilies; or even the differentiation of the N-terminal conformation across different species. Detailed analyses of the modes of motions accessible to the members of the dataset and their variations across members demonstrate how the active and inactive states of GPCRs obey distinct conformational dynamics. The collective fluctuations of the GPCRs are robustly defined in the active state, while the inactive conformers exhibit broad variance among members.

## Introduction

G-Protein Coupled Receptors (GPCRs) are one of the most important protein families involved in signal transduction. They are the largest family of membrane proteins that bind to extracellular molecules which induce a conformational change in the GPCR and allosterically allow for binding of a heterotrimeric G protein or other signaling proteins such as arrestin at the cytoplasmic face of the GPCR.^1–4^ GPCRs are also an extremely important class of drug targets. It is estimated that there are ~700 FDA-approved drugs that target GPCRs^5^; this is around 35% of all FDA approved drugs,^5^ and GPCRs themselves account for 12% of approved drug targets. As this is such an important family of proteins, the development of allosteric compounds would be greatly beneficial because they allow for a greater level of specificity by selectively targeting specific protein–protein interaction sites thereby modulating the specific pathways associated with those interactions. This is in contrast to orthosteric drugs that indiscriminately bind the highly conserved functional site and therefore simultaneously target many family members that share the same orthosteric site and affect multiple pathways.^6–12^

All six classes of GPCR classes (Class A-F) contain a transmembrane (TM) domain composed of seven helices TM1-TM7. Class A GPCRs, also known as rhodopsin-like GPCRs, form by far the largest group, accounting for 85% of GPCRs. They are the most widely studied class, in terms of structure and functional characteristics with a total of about 550 structures resolved either in active or inactive states. While some classes (e.g., B and C) have larger extracellular domains, Class A GPCRs are mainly composed of the TM domain. In all GPCRs, the TM domain is decorated by extracellular (EC) and intracellular (IC) loops having important functional roles, such as the IC loop 3 (ICL3) that undergoes a conformational change during the activation of the receptor and the EC loops that sense external signals. Most of the current drugs target the class A GPCRs and the corresponding diverse receptor families bind different endogenous ligands.

In this paper, we analyze the *signature dynamics* of Class A GPCRs. The signature dynamics of a protein family refers to intrinsically accessible conformational motions encoded by the shared architecture of the family.^13–14^ These motions are represented by the most cooperative motions, often referred to as low frequency or slow modes of motion, within the spectrum of normal modes evaluated using elastic network models.^4,15^ Dissection of the signature dynamics of a family can aid in identifying the critical sites or structural elements that modulate the allosteric behavior shared by all family members as well as the subfamily-specific sites that could be targeted for selectively targeting subfamilies.^13^

## Materials & Methods

### Structural dataset generation

We used the GPCR Database (gpcrdb.org)^16^ for obtaining information on structurally resolved Class A GPCRs. The corresponding structural data were downloaded from the Protein Data Bank (PDB)^17^ using the *ProDy* method *parsePDB()*. The PDB structure (PDB: 7DHI)^18^ resolved β2-adrenergic receptor (β2AR), a GPCR target for treating asthma and chronic obstructive pulmonary disease, was selected as a reference onto which all other Class A GPCRs were structurally aligned using the ProDy function *buildPDBEnsemble()*. GPCR structures that could not be mapped onto the reference, either due to low residue occupancy or missing residues, were removed from the ensemble, which left us with a final set of 160 structures.

Supplemental Table S1 lists the final members of the ensemble of class A GPCRs included in this study, including their PDB ID, their gene codes and corresponding protein names (in the footnote), the PDB chain used here for sequence-structure mapping, the species the GPCR is from, and GPCRdb annotated functional state (active or inactive). The dataset contains structures for 25 unique proteins (each encoded by a different gene) belonging to five different species (squid, bovine, human, rat and mouse) resolved under different conditions (in the presence of different ligands/proteins), e.g. there are 53 rhodopsin structures (gene name *OPSD*), including 5 from Japanese flying squid and 48 from bovine, of which 21 are in inactive conformation and 32 are in active conformation; or 9 muscarinic acetylcholine receptors M2 (gene name *ACM2*), all human, composed of five active and four inactive receptors. The GPCRs are serially ordered according to their sequence similarities as will be described below.

### Methods and metrics for sequence, structure, and dynamics comparisons

Three types of pairwise similarities were measured between the GPCRs in our dataset: sequence, structure, and dynamics similarities. As a metric of sequence dissimilarity, we used the sequence distance given by the (normalized) Hamming distance, defined as the difference [1-*f*] where *f* is the fraction of identical amino acids between the sequentially aligned proteins. The Hamming distance was evaluated using the *ProDy*^19^ function *buildSeqidMatrix()* following the multiple sequence alignment (MSA) of the ensemble using the *getMSA()* function. Structural similarity was measured based on the root-mean-square deviation (RMSD) between the Cα coordinates after optimal structural alignment onto reference structure. The ensemble of structures was subjected to principal component analysis (PCA) using *ProDy* to elucidate dominant changes in structure along two principal coordinates, PC1 and PC2.

The equilibrium dynamics of each structure was characterized using the Gaussian Network Model (GNM).^20–21^ The GNM modes were mapped onto those of the reference β2AR structure using the *SignDy* module^14^ of *ProDy*. The extent of similarity between the structural dynamics of GPCR pairs was assessed by computing their spectral overlap,^22^ also known as covariance overlap, implemented in *ProDy.* The spectral overlap, $SO_{ij}(A,B)$, for a given pair of proteins *A* and *B* provides a measure of the similarity between the spectrum of GNM modes of motion intrinsically accessible to these two proteins, based on the subset of i≤k≤j modes. Higher overlap means higher similarity between the structural dynamics of the two proteins. As a metric, we adopted the spectral distance, defined as the arccosine of the spectral overlap, i.e. $d_{ij}(A,B)=\cos^{-1}\left(SO_{ij}(A,B)\right)$, with lower distance indicating a higher similarity between the mode spectra and *vice versa*.

## Results and Discussion

### Heatmaps for pairwise sequence, structure and dynamics similarities distinguish the species, type, and functional state of the GPCRs

**Figure 1.**
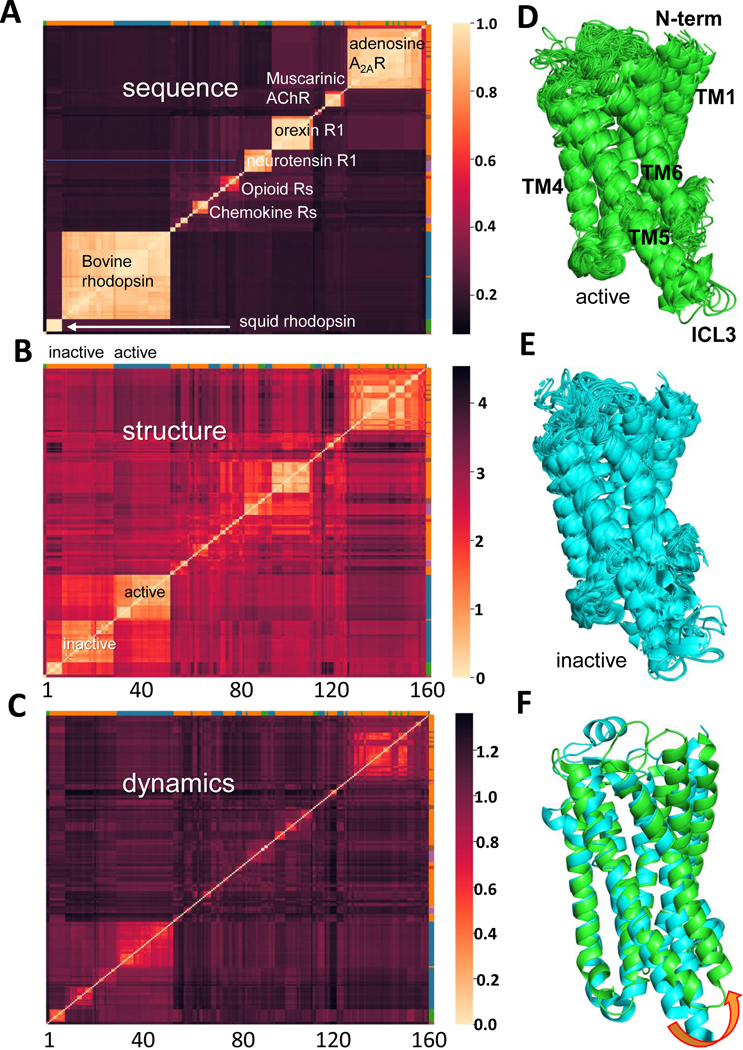
Comparison of GPCR ensemble members by sequence, structure, and dynamics. Heat maps displaying pairwise similarities between GPCRs sequences (**A**), structures (**B**) and dynamics (**C**), are presented. The abscissa and ordinate represent each an individual GPCR using the serial number in Table S1 where they are listed based on their sequence similarities/clusters as shown in panel **A**. The *colored bar* along the right ordinate in each heat map shows which species the GPCR belongs to: *orange* – human, *blue* – bovine, *green* – squid, red – mouse, and *purple* – rat. The *colored bar* along the top abscissa shows which state that structure is in: *blue* – active, *orange* – inactive, and *green* – no label. The sequence similarities of GPCRs in the dataset are measured by sequence identity fraction after sequence alignment (see the *color-coded bar* on the right of panel **A** from 0 to 1 (identical sequences)). In panel **B**, pairwise similarities of structures are measured by RMSD (Å) in Cα coordinates, and in panel **C**, spectral distances are used for measuring the similarities in the equilibrium (structural) dynamics of each pair of GPCRs. Higher values in both structure and dynamics metrics means more dissimilar pairs. Panels **D** and **E** display the superposition of inactive (**D**) and active (**E**) structures, and panel **F** displays a comparison of a representative structure (CXC chemokine receptor resolved by cryoEM) in the active state (PDB ID 6LFO, in *green*) and another from the same ssubfamily (crystal structure of CC Chemokine Receptor 2A) in inactive state (PDB ID 6GPS, in *cyan)*. The curled arrow shows the opening of the cytoplasmic ends of helices TM5–6, connected by the ICL3, upon activation.

As a first step, we examined the degrees of sequence, structure, and dynamics similarities between all pairs of GPCRs in our dataset. The three categories of results are presented as heat maps in the respective panels A-C of Figure 1. In each case, the two axes list the index of the GPCRs (Supplementary Table S1), organized in clusters, based on their sequence similarities. The same sequence-similarity-based order of GPCRs is adopted in all panels to allow for comparisons across sequence, structure, and dynamics spaces. The entries in the respective heat maps **A-C** represent the sequence identity fraction, or (1– Hamming distance) between the sequences of each pair of GPCRs (panel **A**), the RMSD (in Å) between the pairs of structures after optimal structural alignment (panel **B**) and the spectral distances (panel **C**) (see the scales on the *right* of each heat map). In all cases lighter colors represent shorter distances/differences or more similar pairs. Clustered blocks are clearly distinguished in the sequence distance map (Figure 1(A)) (consistent with the ordering of the GPCRs based on their sequence similarities), but they are also identifiable in the structure and dynamics similarity maps, consistent with the mapping ‘sequence → structure → dynamics’, i.e., the fundamental concept of sequence encoding the structure, which, in turn, defines the dynamics.

Examination of Figure 1(A) shows that the lower-left large cluster is mainly composed of bovine GPCRs (rhodopsins) (the color-coded bar along the *right ordinate* refers to different species; see caption), preceded by a small group of rhodopsins belonging to squids. The color-coded bars along the upper abscissa distinguish the active (*blue bars*) and inactive (*orange bars*) forms. Careful examination of this same cluster (of bovine GPCRs) in panel **B** reveals its division into two subclusters, clearly indicating that the active and inactive GPCRs have distinct structures. Notably the active GPCRs show a more coherent conformational dynamics in panel **C**, which apparently reflects their shared mechanism of function in the active state; whereas inactive forms exhibit a variation, as they do not necessarily obey well-defined motions that would otherwise be constrained by functional requirements. The structures of active and inactive GPCRs in the ensemble are shown in Figure 1, panels D and E respectively, and panel F displays the superposition of two representative members to better visualize their structural differences, which will be further elaborated below.

Panel **A** therefore clusters the GPCRs based on their functional types (see a few labels) or by their species when it is the same type (e.g., squid and bovine rhodopsins); whereas panel **B** provides a further discrimination between the active and inactive conformers for a given protein in a given species. Similar effects can be discerned in other regions of the heat map. For example, the upper-right cluster is mainly composed of inactive conformations of human adenosine ${\text{A}}_{2\text{A}}$ receptors (gene *AA2AR* in Table S1). This cluster is subdivided into structural subclasses in panel **B**, and even smaller subsets that obey highly similar dynamics in panel **C**, which presumably reflect the heterogeneities of the inactive forms. A more detailed analysis of the significance of different clusters is presented next.

### The two principal variations in structure account for the activation and speciation of GPCRs, respectively

**Figure 2.**
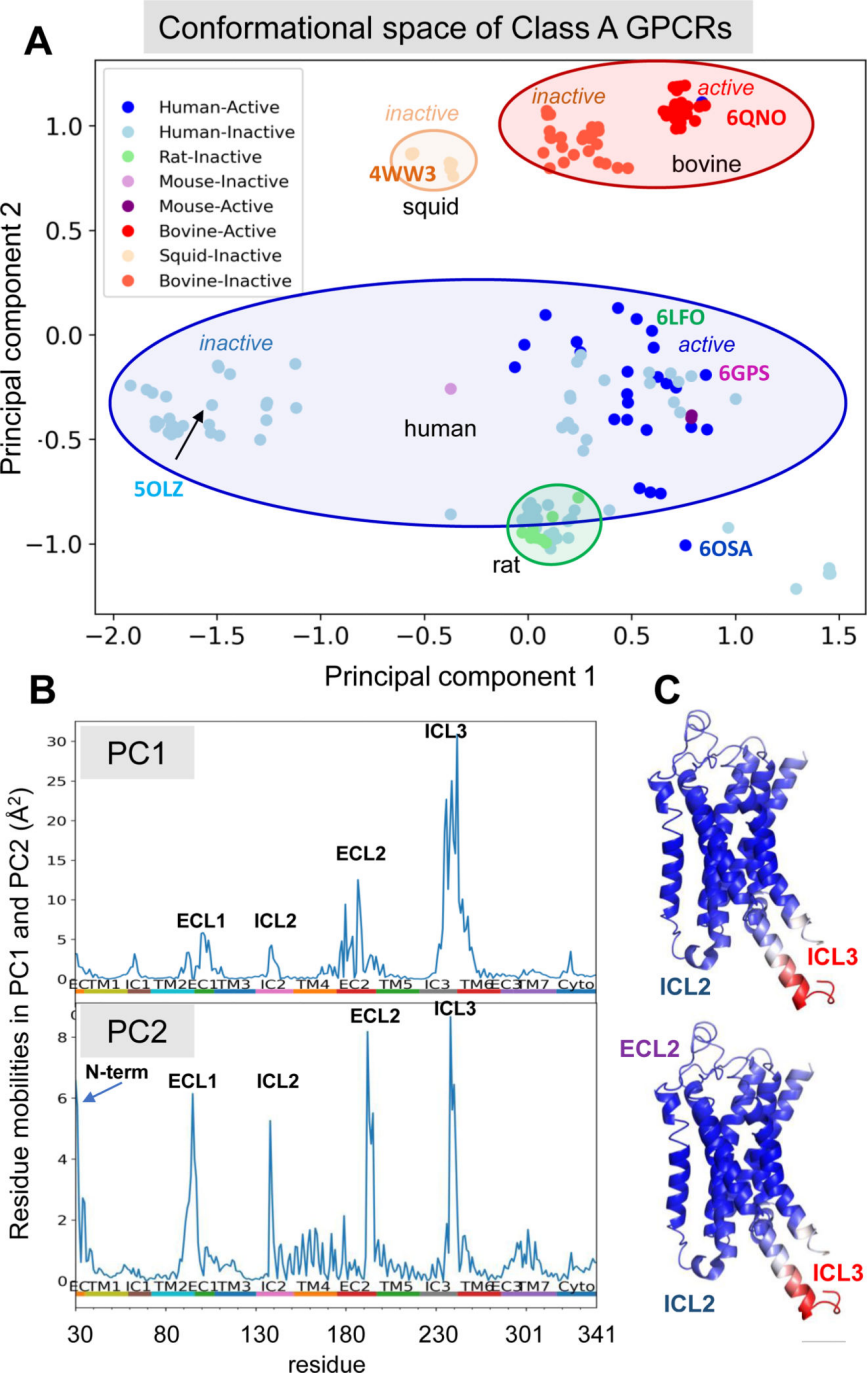
Structural landscape of GPCR ensemble members projected onto a reduced space. Projection of GPCR class A structural ensemble onto the reduced conformational space spanned by the two principal components (PC1 and PC2) of structural variations obtained upon PCA of the ensemble of 160 structures – **A**. Each dot represents one distinct structure. Dots are colored by species (e.g. *blue* – human, *red* – bovine, etc, as indicated by ellipses) and shaded by active state (*dark* – active & *light* – inactive). In panel **B**, the mobility of each residue along the principal coordinates PC1, PC2 and PC3 are plotted. *Colored bars* along the abscissa indicate the structural elements (helices or loops). Residue numbers refer to those of reference structure, β 2AR (PDB id 7DHI). The residues interacting with orthosteric ligand are shown by black dots. **C**. Ribbon diagram color-coded (from *blue* (low) to *red* (high) to illustrate the extent of structural change along PC1, using the reference structure. **D**. The orthosteric ligand (*gray spheres*) and five different allosteric ligands (*sticks in different colors*, see details in text) are shown after alignment on the reference structure. Supplemental Figure S1 displays the counterpart of panel **B** where residues binding those allosteric ligands are shown by dots color-coded by those shown in this ribbon diagram.

We performed a PCA of the dataset of 160 GPCR Class A structures and projected them onto the subspace spanned by the first two principal components PC1 and PC2. This conformational subspace is purely defined by the experimental structural data. It exhibits the types of dominant structural variabilities among the members of the examined dataset. PC1 and PC2 refer to the principal directions (variables encompassing the collective displacement of all residues) associated with the largest variations in structures. Of interest is to see where the individual GPCRs in our dataset localize in this subspace. The result is shown in Figure 2(A). Each dot therein represents one GPCR (listed in Table S1). The dots are color-coded by species as labeled, and *light* and *dark tones* of the same color refer to the active and inactive GPCRs corresponding to that species. A clear separation of the GPCRs based on the species to which they belong (human (*light/dark blue dots*) and bovine (*light/dark red dots*) or squid (*orange*), or even rat (*green*)) is achieved along PC2, as indicated by the *color-coded ellipses*.

The first principal component PC1, on the other hand, shows a clear split between active (*red*) and inactive (*orange)* conformations of bovine GPCRs. A similar but less sharpy defined separation between active *(dark blue*) and inactive *(light blue*) conformations could be observed within the set of human GPCRs (*enclosed by the blue ellipse*), but the position along PC2 is also affected by specific function of the subfamilies, as will be elaborated below, hence the dispersion of *light blue dots* among *dark blue* ones. It is interesting to note that the mouse active and inactive structures (indicated by *dark* and *light violet* dots respectively, within the human *ellipse)* also display a shift toward positive PC1 direction upon activation.

Therefore, the principal structural variation PC1 accounts for the structural changes involved in the activation or inactivation of the GPCRs, in general, whereas PC2 enables species-specific conformational rearrangements, regardless of being active or inactive. The conformational space thus clearly shows how the two most important collective rearrangements of the GPCRs structures have evolved to enable its allosteric transition between active and inactive forms (PC1) and its speciation (PC2). Movements along PC2 further enable functional differentiation among subfamilies as will be elaborated below.

### Closer examination of the principal variation (PC1) reveals shared activation mechanisms conserved across GPCRs and subfamily-specific features that enable functional adaptability

**Figure 3.**
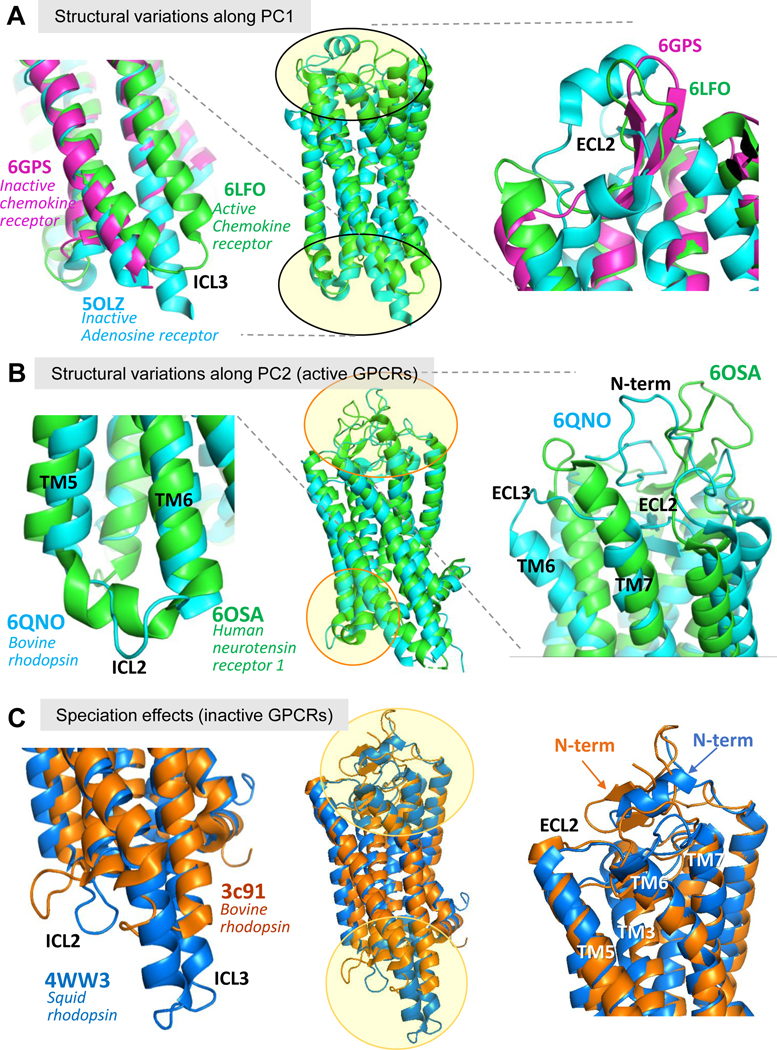
Comparisons of GPCR structures to visualize the principal changes in structures. Structural alignment of active state (*green* – 6LFO) and inactive state (*cyan* – 6GPS & *magenta* – 5OLZ) GPCR structures – **A**. Whole structural alignment is shown in the central panel, with zoomed in view of the areas with highest variability along PC1, *left* – ICL3 and *right* – ECL2. Panel **B** shows a similar alignment showing the variations along PC2. The aligned structures are human neurotensin receptor 1 (*green*-6OSA) and bovine rhodopsin (*cyan*-6QNO) both in the active state. Close-up inserts show ICL2 on the *left* and the EC vestibule on the *right*. Panel **C** shows alignment of two rhodophsins from squid (*blue*-4WW3) and bovine (*orange*-3C91).

Residue displacements (with respect to average coordinates) along the two principal directions of collective structural changes PC1 and PC2 are plotted in the respective *top* and *middle* panels of Figure 2(B). PC1 and PC2 account to 30.76% and 13.95% of the total variance in the ensemble. PC3 (11.57%) is also shown in the *bottom* panel, where only a sharp peak is observed at ICL3. The *top* panel shows that the largest displacement in PC1 occurs at the IC loop ICL3 and the cytoplasmic ends of TM helices TM5 and TM6, on both sides of the loop. In fact, ICL3 is not even resolved in many structures (including the reference β2-adrenergic receptor displayed here) due to its high conformational variability. The N-terminal (cytoplasmic) end of the transmembrane helix TM6 and C-terminal (cytoplasmic) end of TM5 (enclosed in the *black circle* and enlarged on the *left diagram* in Figure 3(A); see also Figures 1(F) and 2(C)) are known to participate in the region where the largest conformational shift occurs when the GPCR transitions from its inactive to its active state.

In Figure 2(B), the dots along the curve indicate the location of the residues that coordinate the orthosteric ligands that bind to the GPCRs. The counterparts of these three panels indicating the residues that coordinate allosteric ligands is presented in Supplemental Figure S1. The residues forming the orthosteric site are based here on our reference β2AR structure (PDB 7DHI) bound to the partial agonist salbutamol, which is shown by *dark gray spheres* in Figure 2(D). The orthosteric site residues are mostly located on TM helices and display minimum variability, if any, along PC1. There is only one residue (F193) belonging to the long variable loop ECL2 that interacts with salbutamol, which enjoys a high conformational variability.

While active and inactive conformers of bovine GPCRs are clearly separated along PC1 (Figure 2 (A)), the human subset exhibits a number of structures annotated as inactive (*light blue* in panel Figure 2(A)), which are mixed in with active conformation structures (*dark blue*) along this axis, as mentioned above. We examined what type of conformational changes gave rise to this interspersing of inactive conformers among the active ones. Our examination revealed that this ‘mixing’ mainly originated from the conformational variability in the extracellular loop 2 (ECL2). Figure 3(A) compares the structures of an active state GPCR (CXC chemokine receptor; PDB 6LFO; *green ribbon diagram*) and two inactive structures, one near the active structure (CC chemokine receptor 2A, PDB 6GPS; *magenta*) and the other far (adenosine ${\text{A}}_{2\text{A}}$ receptor; PDB 5OLZ, *cyan*) along PC1 (see Figure 2(A)). We see that in both inactive structures the TM5-ICL3-TM6 region is clearly shifted inwards compared to the active state structure (*left panel* in Figure 3(A)).

However, the *right panel* shows that the ECL2 of the inactive CC chemokine receptor 2A (*magenta*) is much more similar to that of the active state (of CC chemokine receptor 2A; *green*) than the other inactive structure (of adenosine ${\text{A}}_{2\text{A}}$ receptor). This analysis shows that a common structural feature at ECL2 shared by two members of the same subfamily (of chemokines) has determined their close location in the conformational space despite their differences at the TM5-ICL3-TM6 region typical of their different state of activation. The common structural motif (β-hairpin-like) shared by this subfamily members at ECL2, presumably evolved to assist in allosteric binding of their ligands (chemokines) from the EC region.^23^

We note that ECL2 is the longest loop in Class A GPCRs, and exhibits a variety of structural motifs, as recently categorized into seven clusters, clusters A-G, based on their sequence, structure, and intramolecular contacts.^24^ The chemokine receptors shown in *green and magenta* ribbons in Figure 3(A) belong to cluster B, the most populated cluster, where members carry the antiparallel β - sheet (or β-hairpin) shown in Figure 3(A) (*right*). Adenosine receptors, represented in the same panel by the *cyan diagram*, on the other hand, belong to Cluster C, and in this case ECL2 contains a small helix.^24^ The dispersion of structures along the PC1 axis is thus affected by not only the activation/inactivation state of individual GPCRs as determined by the cytoplasmic TM5-ICL3-TM6 region, but also by other structural characteristics, and in particular the ECL2, that often confer the functional specificities of the GPCR Class A subfamilies.

Overall, GPCR subfamily-specific commonalities in local structures, as illustrated here for the ECL2 of two members of the chemokine family, may overshadow the differences in structure near ICL3 (typical of activation) and give rise to the ‘mixing’ of active and inactive conformations as seen in Figure 2(A) *large blue ellipse*. Such commonalities among subfamily members confers their functional specificity and reveal the mechanisms of adaptations that enable a diversity of function by the different subfamilies of GPCR family.

### Structural variations in the loops ICL2, ICL3, ECL1 and ECL2, in addition to those in the N-terminal segment underlie Class A GPCRs’ speciation and specificity

In the case of structural variations along PC2, five sharp peaks are observed as the highest mobility regions (in Figure 2(B), *middle panel*). One of them is again the ICL3 connecting the cytoplasmic ends of TM5-TM6, corroborating that the same type of activation mechanism occurs across species. Two of the other peaks are at the EC loops ECL2 and ECL1, and the adjoining helical ends, ECL2-TM5 and TM2-ECL1; and the last two are at the IC loop ICL2 and at the N-terminal segment.

Figure 3(B) compares two structures bovine rhodopsin and human neurotensin receptor type 1 (respective PDB ids 6QNO and 6OSA). These are selected as two structures located at the opposite ends of the PC2 axis (that differentiates the species; see Figure 2(A)), while being at the same position along PC1 so as to discern the structural variations along PC2. They are both active, such that the differences due to activation are not present. The *left* and *right* panels in Figure 3(B) illustrate the structural differences taking place at the ICL2 and EC vestibule, respectively. The differences at the EC region, and in particular in ECL2, are extensive, indicating the role of this region in recognizing species-specific ligands, in addition to subtype-specific ligands. At the ICL2 region, we see that the human neurotensin receptor 1 (*green*) loop is in the characteristic α-helical structure of this loop in active state, whereas the bovine rhodopsin (*cyan*) ICL2 has no secondary structure. In the EC vestibule (*right*), the EC2 loop antiparallel β-strands occupy different positions, one is exposed, the other is buried deeper into the EC vestibule. We can also see a structural rearrangement at the N-terminal segment. We note that the N-terminal structural variations are not included in our ensemble analysis as this 30-residue segment was not resolved in many GPCRs (being highly flexible), including our reference structure.

In order to further view the effect of speciation on the structure, in the absence of GPCR type (or subfamily) specific effects, we further compared the structures of the bovine and flying squid rhodopsins, both inactive state (respective PDB ids 3C9L and 4WW3). The bovine rhodopsin is shown in Figure 3(C) in *orange*, and the squid in *blue*. Comparison of this pair of structures reveals again major differences at their N-terminal segments, corroborating the role of the N-terminal segment in discriminating different class A GPCRs. Furthermore, we observed structural variations in the loops ICL2, ICL3 and ELC2, consistent with the peaks observed in the PC2 profile (Figure 2(B) *middle*).

Overall, this analysis further supports the involvement of the principal modes PC1 and PC2 in the activation, subfamily-specific adaptations and speciation of Class A GPCRs.

### Allosteric sites exhibit site-specific variability in line with the GPCR intrinsic dynamics

Residues that coordinate the allosteric ligands (displayed in Figure 2(D)) are indicated by *color-coded dots* on the PC1-PC3 profiles presented in Supplemental Figure S1. There are numerous allosteric sites resolved for Class A GPCRs. The residues interacting with five different allosteric ligands were determined using alternative structures from our dataset and displayed in Figure 2(D). Allosteric ligands bound to exosites (allosteric sites that affect activation when occupied) are taken from the structures resolved for an active human M2 muscarinic acetyl choline receptor (PDB 4MQT; *black sticks*) and a rat succinate receptor complexed with an antagonist (PDB 6NRK; *red sticks*). These exosites are located above the orthosteric site, thus interact with the upper helices and extracellular loops. Two other allosteric ligands with lipid-facing orientation are extracted from the structures resolved for the human P2Y1 receptor (PDB 4XNV; *green sticks*) and human C5 anaphylatoxin chemotactic receptor (C5AR) (PDB 5O9H; *purple sticks*), as well as another GPCR, human cysteinyl leukotriene receptor that is partially lipid-facing (PDB 6RZ9*; light orange* sticks). In contrast to the orthosteric sites which exhibited minimal conformational variability, most allosteric sites show variability along both PC1 and PC2. The highest variations are observed for allosteric ligand-binding residues located on ECL1 and ECL2. The allosteric ligand bound to C5AR (*purple*) at the middle portion of the membrane interacts only with helices (TM3, TM4 and TM5), therefore its variability is observed in tandem with that of TM4 along PC2.

### Signature dynamics encoded by the 7-TM architecture is a major determinant of ensemble behavior

The above analysis provides information on the conformational landscape accessible to the ensemble of Class A GPCRs, and on the functional significance of the two PCs along this landscape. This information is purely based on the statistical analysis of structural data resolved to date. We further investigated the conformational dynamics intrinsically accessible to Class A GPCRs. The intrinsic dynamics refers to equilibrium fluctuations in conformations that are uniquely defined or encoded by the fold. Examination of the intrinsic dynamics of protein families in recent work demonstrated how the global (most cooperative, lowest frequency) modes of motions (e.g. *modes 1–3*) predicted by elastic network models are shared by family members and define the signature dynamics of the family, whereas those in the low frequency (LF) and low-to-intermediate frequency (LTIF) regimes (respective *modes 4–20*, and *21–60*, respectively) reveal subfamily specific features.^14^ These subsets of modes define the signature dynamics of the family and its differentiation among subfamilies. Of interest is to examine how the theoretically predicted modes of movements accessible to Class A GPCRs in different mode regimes relate to the PCs derived from the experimental dataset.

**Figure 4.**
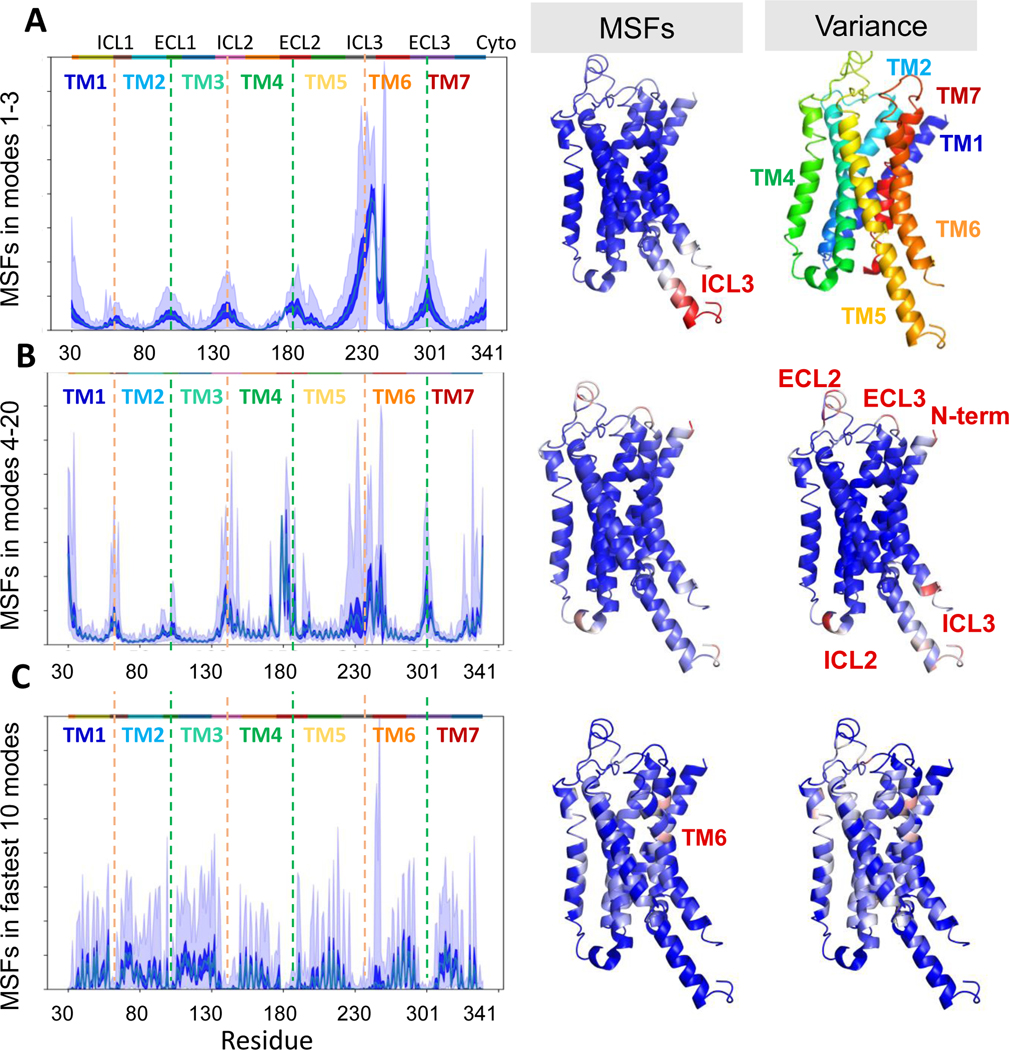
GPCR signature dynamics in specific mode regimes. *Left* panels show the signature GNM profiles, or the distributions of the mean-square fluctuations (MSFs) of residues corresponding to specific mode regimes, (**A**) slowest modes 1 to 3, (**B**) modes 4 to 20, and (**C**) fastest 10 modes, as calculated using the *SignDy* module^14^ of *ProDy*^41^. The average value over all ensemble members at each residue is shown by the *thin green curve* in each plot. The *dark blue shaded* area (which eclipses the *thin green curve*) shows the variance at each residue, and the *light shaded* area shows the minimum and maximum values at each position. The average values and the variances corresponding to each panel are shown mapped onto the color-coded β 2AR reference structure in the *middle* and *right* panels, respectively, with *blue-to-red*-colors representing *rigid-to-mobile* regions.

To this aim we analyzed the fluctuations of residues driven by different subsets of GNM modes and their variances among family members. Figure 4 panels A-C display the mode shapes (mean-square fluctuations (MSFs) of residues) in different mode regimes, showing their variance among members of the family (*dark blue shades*) and their maxima/minima (*light blue shades*). The average fluctuation profile of the ensemble is represented by *thin green line*s, which are eclipsed by the *dark blue shades* on each panel. For each of these mode regimes, the average MSFs (average over the ensemble) as well as the variance in the square fluctuations are mapped onto the color-coded ribbon diagram of b2AR that has been used as reference. Colors on ribbon diagrams refer to size of fluctuations, from low (*blue*) to high (*red*). Global modes (*modes 1–3; panel* **A**) account for large, structural changes in protein conformation. As expected, the TM helices show the lowest amount of overall motion and the lowest variance (minima in Figure 4(A)). The LF (*modes 4–20,* Figure 4(B)) and LTIF (modes *21–60,* not shown here) regimes show a similar distribution, with TM helices exhibiting largely restrained and conserved dynamics, and intra/extra-cellular loops showing greater flexibility and variability in dynamics.

The major difference between the panels **A** and **B** of Figure 4 lies in the relative mobilities of the loops (in particular, the loops ICL3 and ECL2) and chain termini. For visual clarity the positions of the EC and IC loops are indicated by the respective *green and orange dashed vertical* lines. In the case of the global modes (panel **A**), by far the greatest amount of flexibility and variance is predicted in the ICL3. This is consistent with the dominant structural change observed in experiments (Figure 2(B-C) & Figure 3(A)). This is the loop between TM5/6 which undergoes a conformational shift when switching between active and inactive conformations, as discussed above. This loop also has a large amount of variability in its length and sequence between different GCPR Class A members which would help explain the large difference in dynamics observed. In the low frequency regime (LF, *modes 4–20*, Figure 4(B)), we see that in addition to ICL3, all other extracellularly and intracellularly exposed helical ends and loops (except ECL1) as well as the N-and C-termini exhibit large and almost equally distributed fluctuations. The same regions also show the largest variations among family members (*rightmost ribbon diagram* in panel **B**). Similar patterns are also observed in the LTIF regime (not shown), where the differences between different loops are further minimized, and peaks and minima retain their respective positions at loops (or termini) and central portions of the TM helices.

The LF and LTIF regimes have been shown to involve modes of motions that underlie the functional selectivity or dynamic differentiation of protein family members.^14,25^ The large variability in the structural dynamics of ECL2 and ECL3 as well as N-terminus in this regime suggests that these variabilities refer to the specificities of family members, consistent with the above ensemble analysis. In fact, these loops have previously been observed to aid in receptor selectivity of a number of GPCRs.^26–28^

The highest frequency modes (highest frequency 10 modes of each structure; Figure 4(C)) exhibit a completely different behavior: peaks are located at the most closely packed (central) regions of TM helices, while minima are at EC- or IC-exposed regions. The profile also shows a high variability among family members, being sensitive to amino acid substitutions. High frequency GNM modes point to folding nuclei, or regions that are evolutionarily conserved and confer stability.^29^ The peaks here indeed correspond here to the core of the TM domain, and includes the counterparts of the residues identified earlier in rhodopsin as core amino acids.^30^

Finally, we analyzed the global modes predicted by the anisotropic network model (ANM) using our reference β2AR structure (PDB: 7DHI). The Supplementary Movie 1 panels A-D show the collective motions along the lowest frequency four modes, shortly designated as ANM1-ANM4. Comparison with PC1–3 showed that the second ANM mode, ANM2, exhibits a relatively high correlation (0.66) with PC1 direction. Note that we are comparing two 3 *N*-dimensional vectors (PC1 and ANM2 for *N* > 300 residues) and a correlation cosine of 0.66 is higher than that expected from random (1/3*N*) by a factor of ~600. Furthermore, our analysis showed that ANM1 (see Supplementary Movie 1A) correlates with PC3 by a correlation cosine of 0.67. Finally, among the top 20 ANM modes, one (ANM15) turns out to give a correlation of ~0.20 with PC2, which is still two orders of magnitude higher than random.

The high correlation between PC1 and ANM2 reveals that the structural changes involved in activation, derived from the ensemble analysis of all GPCRs in our dataset, could be traced back to the structural dynamics of individual GPCRs encoded by their specific 7-TM architecture, as demonstrated here by the ANM analysis β 2AR. Previous ANM analysis^31^ and MD simulations guided by normal modes^32^ indeed showed the pre-disposition of Class A GPCRs to allosterically undergo the required functional changes at the TM5-ICL3-TM6 cytoplasmic region coupled to chromophore binding. Furthermore, despite the incomplete structure at the ICL3, the motions predicted here closely reproduce those observed in a recent ANM analysis for β 2AR structural models containing the ICL3, as well as those sampled in full atomic MD simulations, both conducted in the presence of membrane.^33^ The robustness of global ANM modes (or insensitivity to missing short segments) has been pointed out in several studies in the last two decades, since the introduction^20,34^ of the elastic network models.

### Comparison of the global dynamics of active and inactive GPCR conformations

**Figure 5.**
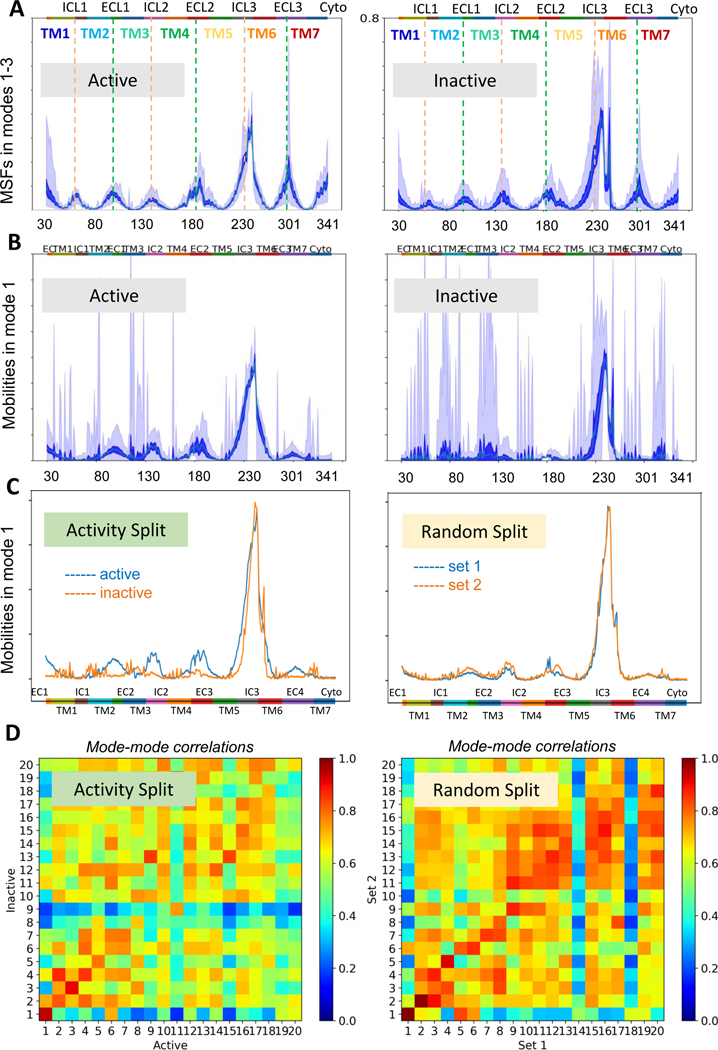
Intrinsic dynamics of active and inactive state GPCR ensembles, in comparison to that of randomly split ensembles. (**A**) Comparison of the global dynamics (modes 1–3) of the active (left panel) and inactive (right panel) subsets of Class A GPCRs. (**B**) Same as (**A**), focusing on mode 1 only. (**C**) Significance of the difference between the 1**st** mode accessible to the active and inactive GPCRs, estimated upon comparison to the difference between two randomized subsets. The ***left panel*** shows the results for ensembles split by *blue* – active vs *orange* – inactive states; whereas the right panel shows plots for two randomly selected subsets where the differences practically vanish. (**D**) Correlation cosines between slowest modes accessible to the active and inactive Class A GPCRs *(left heatmap*) and those accessible to randomly selected subsets (*right heatmap*) profiles of different structural ensembles were calculated and plotted as heatmaps. The former shows lower correlations, indicating the differences between active and inactive GPCRs. See more details in Supplementary Figures S2 – S4.

After splitting the dataset into active and inactive subsets based on the labels in the GPCRdb, *SignDy* analysis was performed again for the two subsets, to compare their signature dynamics (Figure 5(A)). A clear difference we observed was the much greater amount of variance in the dynamics of the global modes of ICL3 in the *inactive* ensemble compared to the active ensemble. This would imply that in the active state, Class A GPCRs largely share the same global modes of motion, even in the TM5/ICL3/TM6 region which accounts for the largest amount of overall motion in the structure. Activation (upon ligand binding) eliminates much of the disorder/variance at the TM5/ICL3/TM6 region, presumably resulting in a well-defined functional configuration amenable to intracellular substrate binding. A larger overall level of variance is observed in the inactive state in other modes as well (Supplementary Figure S2), compared to the modes accessible in the active state, even though this effect is not as pronounced as the global modes. Overall, the active forms have less disorder or conformational diversity than the inactive forms throughout the entire mode spectrum. This reduction in variance/disorder, especially at the global modes (that would be expected to have high variance accompanying their larger size movements), points to the evolutionary optimization and conservation of cooperative movements that ensure activation for all GPCR Class A members.

To obtain a better understanding of the differences in dynamics between active and inactive conformations, individual global modes (modes 1–3) for each ensemble were visualized using *SignDy* (Supplementary Figure S3). For mode 1 (Figure 5(B)), we see two distinct differences between the active and inactive ensembles. First, structures in the inactive conformation have a much higher variance in dynamics compared to those in the active conformation, especially in the ICL3/TM6 area. Second, while the variance is higher in the inactive ensemble, the MSFs in the active ensemble are higher in the ECL1/ICL2/ECL2 loops. This further suggests a greater level of cooperativity of motion for structures in the active conformation. Whereas the dynamics of the first mode in the inactive ensemble is dominated by the ICL3/TM6 region, structures in the active state have enhanced movements more spread out in significant regions throughout the protein structure.

To validate that this difference in ensemble dynamics is significant, a second set of structural ensembles was analyzed by randomly selecting members and repeating the *SignDy* analysis as previously described. MSF values for individual modes were calculated for these new ensembles and compared to the corresponding modes from the active/inactive ensembles. Figure 5(C) shows the results for mode 1, and Supplementary Figure S4 for each of the first three modes. As expected, while the overall mode shapes are similar between the active/inactive ensembles and the randomly selected ensembles, the difference between the active and inactive subsets originally observed (shown in the *left panels*) completely disappear when the average behaviors of two randomly selected subsets are compared (*right panels*).

Figure 5(D) compares the mode–mode correlations between the active and inactive GPCR subsets (left) and those in the randomly selected Sets 1 and 2. In these heat maps the overlaps between individual modes (modes 1–20) are shown for the active and inactive ensembles (*left map*), and the randomized split (*right map*). Notably, even though a few slow modes show strong correlations in either pair of subsets, the differences between active and inactive are much greater compared to the differences between subsets 1 and 2, again pointing to the distinctive behavior of the active and inactive conformers. Likewise, the standard deviations in those mode–mode correlations are much smaller in the active/inactive pair, compared to the randomized split. These observations highlight the distinct dynamics of the active and inactive forms (smaller overlap in MSFs), and the robustness of their dynamics (smaller standard deviations) compared to the randomized sets.

## Conclusion

In this study, we expanded on our previous work on ensemble analysis of signature dynamics of protein families for investigating the structure–function relations in Class A GPCRs. This class of GPCRs are among the most important targets of current drug compounds. Compounds that allosterically modify the activity of GPCRs of interest are beneficial because they are by far less likely to result in off-target effects than compounds that target orthosteric sites. To this end, we presented detailed ensemble analyses of sequence, structure, and dynamics properties of a dataset of Class A GPCRs and compared the ensemble behavior with the collective motions predicted by elastic network models. While sequence alignments differentiated different types and species of GPCRs, structural alignments provided a further discrimination of active and inactive states for a given species and type, which were further subdivided into subgroups based on heterogeneities in the intrinsic dynamics.

The analysis elucidated major structural changes that occur between family members belonging to different species (along PC2) as well as those involved in activation (along PC1) and how EC or IC loops’ rearrangements make significant contributions to both speciation and subtype specificity. As observed in prior studies, the TM5/ICL3/TM6 region showed the largest amount of conformational flexibility. It also exhibited the largest variance in dynamics between family members. This makes sense as the shifting outward of this region is a characteristic feature of GPCR activation, with this opening allowing access to the G protein binding site on the cytoplasmic face of the protein. Indeed, when splitting the Class A subfamily into subsets based on their label for ‘active’ or ‘inactive’ in the GPCRdb^16^ we saw a distinct difference in dynamics of the global modes between these two subsets. These differences are largely confined to the TM5/ICL3/TM6 region, however there is also a notice-able difference in dynamics of the ECL2 loop. This loop has previously been observed to be involved in binding of both GPCR orthosteric-site and allosteric-site targeting compounds.^23,35–36^

Interestingly, ECL2 loop was also found to be significant when analyzing the structural ensemble using principal component analysis. While bovine GPCRs were divided clearly into active and inactive groupings along PC1, human GPCRs showed a cluster of active state structures in the middle of PC1 with several inactive structures intermixed. An analysis of these structures showed that while the TM5/ICL3/TM6 region of these ‘inactive’ labeled structures was indeed in the inactive or closed conformation, the ECL2 loop showed a much greater similarity with the ECL2 loop of active conformation GPCRs. Thus, classification of the active and inactive forms into two structural subclasses is a simplification, given the complexity of the structures and heterogeneity of conformational changes on a local scale. Yet, the two functionally distinct group were distinguished by their distinctive dynamics. The significance of these differences has been demonstrated by repeating the analysis for randomly selected subsets.

Importantly, conformational heterogeneities represented by the ensemble PCs were shown to correlate with the signature dynamics of the family or with the global/slow ANM modes intrinsically encoded by the evolutionarily optimized 7-TM architecture. These modes, grouped in two regimes (global, modes 1–3; and slow, modes 4–20) are likely to favor, if not enable, the structural changes associated with speciation, subtype specificity (within a given species), or activation (of a given subtype). It is conceivable that an ensemble of GPCR conformations preexist prior to a population shift induced by ligand binding.^37^ Alternatively, individual GPCRs themselves may have sufficient dynamic flexibility to fluctuate between multiple conformers along these ‘soft’ modes (that require small energy ascent). This type of dynamic flexibility, or ‘conformational stochastics’ (as opposed to the statistical thermodynamics view of populations) may help explain the continuous landscape of conformations, or the occurrence of partial agonisms and observed dynamic range of G protein activation.^38^ Further analysis toward understanding the distinct (soft) modes of motion that facilitate allosteric changes in favor of full, partial, reverse, or biased agonism (i.e., arrestin binding) may help design allosteric modulators with selective actions.

A goal for further analysis based on this research is to combine this family-level analysis with analysis of local ligand binding probability such as druggability screening^39^ or essential site scanning (ESSA).^40^ This analysis would potentially allow targeting of drug screening efforts to regions that would affect the dynamics of specific proteins while avoiding regions with highly conserved dynamics, possibly improving drug specificity while reducing off-target effects. The same analysis could be extended to other classes of GPCRs as well. Class A GPCRs are unique among GPCR members as they contain only the characteristic transmembrane domain; other classes contain significant extracellular domains as well. These would potentially make ensemble analysis more challenging, with structural alignment specifically being a significant challenge given the flexible connection between transmembrane and extracellular domains.

## Supplementary Material

Table S1

Supplementary figures

Supplemental Movie 1

## Data Availability

Data will be made available on request.

## Abbreviations:

GPCR: G protein coupled receptor
ENM: elastic network model
ICL3: intracellular loop 3
GNM: Gaussian Network Model
EC: extracellular
SO: spectral overlap
PDB: Protein Data Bank
