# Supplementary material for: Activation and Speciation Mechanisms in Class A GPCRs
Source: J Mol Biol. Author manuscript; Available in PMC 2023 Apr 25. (PMC10129049; doi:10.1016/j.jmb.2022.167690)
Supplement: Table S1 [file NIHMS1881903-supplement-Table_S1.docx]

Supplemental Table S1. Members of GPCR Class A ensemble included in the present analysis

| Protein index | PDB ID | *Gene^(a)^* | Chain in the PDB file | Species | State in the resolved structure |
| --- | --- | --- | --- | --- | --- |
| 1 | 2ZIY | *OPSD* | A | Japanese flying squid | Inactive |
| 2 | 2Z73 | *OPSD* | A | Japanese flying squid | Inactive |
| 3 | 4WW3 | *OPSD* | A | Japanese flying squid | Inactive |
| 4 | 3AYM | *OPSD* | B | Japanese flying squid | Inactive |
| 5 | 3AYN | *OPSD* | B | Japanese flying squid | Inactive |
| 6 | 5TE5 | *OPSD* | A | Bovine | Inactive |
| 7 | 1F88 | *OPSD* | A | Bovine | Inactive |
| 8 | 1HZX | *OPSD* | A | Bovine | Inactive |
| 9 | 1L9H | *OPSD* | A | Bovine | Inactive |
| 10 | 1U19 | *OPSD* | A | Bovine | Inactive |
| 11 | 2PED | *OPSD* | B | Bovine | Inactive |
| 12 | 2G87 | *OPSD* | A | Bovine | Inactive |
| 13 | 3OAX | *OPSD* | A | Bovine | Inactive |
| 14 | 2HPY | *OPSD* | B | Bovine | Inactive |
| 15 | 2I37 | *OPSD* | A | Bovine | Inactive |
| 6gps | 3C9L | *OPSD* | A | Bovine | Inactive |
| 17 | 1GZM | *OPSD* | A | Bovine | Inactive |
| 18 | 2I35 | *OPSD* | A | Bovine | Inactive |
| 19 | 2I36 | *OPSD* | A | Bovine | Inactive |
| 20 | 2J4Y | *OPSD* | B | Bovine | Inactive |
| 21 | 3C9M | *OPSD* | A | Bovine | Inactive |
| 22 | 6CMO | *OPSD* | R | Human | Active |
| 23 | 6QNO | *OPSD* | R | Bovine | Active |
| 24 | 6FK7 | *OPSD* | A | Bovine | Active |
| 25 | 6FK8 | *OPSD* | A | Bovine | Active |
| 26 | 6FKD | *OPSD* | A | Bovine | Active |
| 27 | 6FKB | *OPSD* | A | Bovine | Active |
| 28 | 6FKA | *OPSD* | A | Bovine | Active |
| 29 | 6FKC | *OPSD* | A | Bovine | Active |
| 30 | 6FK9 | *OPSD* | A | Bovine | Active |
| 31 | 6FK6 | *OPSD* | A | Bovine | Active |
| 32 | 6OY9 | *OPSD* | R | Bovine | Active |
| 33 | 4PXF | *OPSD* | A | Bovine | Active |
| 34 | 3CAP | *OPSD* | A | Bovine | Active |
| 35 | 5DYS | *OPSD* | A | Bovine | Active |
| 36 | 3PXO | *OPSD* | A | Bovine | Active |
| 37 | 6OYA | *OPSD* | R | Bovine | Active |
| 38 | 6PEL | *OPSD* | A | Bovine | Active |
| 39 | 6PGS | *OPSD* | A | Bovine | Active |
| 40 | 5TE3 | *OPSD* | A | Bovine | Active |
| 41 | 3DQB | *OPSD* | A | Bovine | Active |
| 42 | 3PQR | *OPSD* | A | Bovine | Active |
| 43 | 5EN0 | *OPSD* | A | Bovine | Active |
| 44 | 4BEY | *OPSD* | A | Bovine | Active |
| 45 | 4BEZ | *OPSD* | A | Bovine | Active |
| 46 | 5WKT | *OPSD* | A | Bovine | Active |
| 47 | 4J4Q | *OPSD* | A | Bovine | Active |
| 48 | 6PH7 | *OPSD* | A | Bovine | Active |
| 49 | 6NWE | *OPSD* | A | Bovine | Active |
| 50 | 4A4M | *OPSD* | A | Bovine | Active |
| 51 | 6FUF | *OPSD* | A | Bovine | Active |
| 52 | 4X1H | *OPSD* | A | Bovine | Active |
| 53 | 2X72 | *OPSD* | A | Bovine | Active |
| 54 | 6RZ6 | *CLTR2* | A | Human | Intermediate |
| 55 | 6RZ9 | *CLTR2* | A | Human | Intermediate |
| 56 | 6RZ7 | *CLTR2* | A | Human | Intermediate |
| 57 | 4XNV | *P2RY1* | A | Human | Intermediate |
| 58 | 4XNW | *P2RY1* | A | Human | Intermediate |
| 59 | 6IBB | *SUCNR1* | A | Rat | Intermediate |
| 60 | 6Z10 | *SUCNR1* | A | Rat | Intermediate |
| 61 | 6RNK | *SUCNR1* | A | Rat | Intermediate |
| 62 | 4XT1 | *US28* | A | Human cytomegalovirus | Active |
| 63 | 6KNM | *APJ* | B | Human | Inactive |
| 64 | 5VBL | *APJ* | B | Human | Inactive |
| 65 | 6GPS | *CCR2* | A | Human | Inactive |
| 66 | 6GPX | *CCR2* | B | Human | Inactive |
| 67 | 5UIW | *CCR5* | A | Human | Inactive |
| 68 | 6AKY | *CCR5* | A | Human | Inactive |
| 69 | 6AKX | *CCR5* | B | Human | Inactive |
| 70 | 4MBS | *CCR5* | A | Human | Inactive |
| 71 | 6WWZ | *CCR6* | R | Human | Active |
| 72 | 6LFO | *CXCR2* | R | Human | Active |
| 73 | 6LFM | *CXCR2* | R | Human | Active |
| 74 | 6OS2 | *AGTR1* | A | Human | Active |
| 75 | 6OS1 | *AGTR1* | A | Human | Active |
| 76 | 6DO1 | *AGTR1* | A | Human | Active |
| 77 | 6JOD | *AGTR2* | A | Human | Active |
| 78 | 4EA3 | *OPRX* | A | Human | Inactive |
| 79 | 5DHH | *OPRX* | A | Human | Inactive |
| 80 | 5DHG | *OPRX* | A | Human | Inactive |
| 81 | 6VI4 | *OPRK* | B | Human | Inactive |
| 82 | 6B73 | *OPRK* | A | Human | Active |
| 83 | 6DDE | *OPRM* | R | Mouse | Active |
| 84 | 6DDF | *OPRM* | R | Mouse | Active |
| 85 | 5O9H | *C5AR1* | A | Human | Inactive |
| 86 | 6OMM | *FPR2* | R | Human | Active |
| 87 | 4BV0 | *NTR1* | A | Rat | Inactive |
| 88 | 4BWB | *NTR1* | A | Rat | Inactive |
| 89 | 4BUO | *NTR1* | A | Rat | Inactive |
| 90 | 3ZEV | *NTR1* | A | Rat | Inactive |
| 91 | 6PWC | *NTR1* | R | Human | Active |
| 92 | 6UP7 | *NTR1* | R | Human | Active |
| 93 | 6OSA | *NTR1* | R | Human | Active |
| 94 | 6OS9 | *NTR1* | R | Human | Active |
| 95 | 6TOT | *OX1R* | B | Human | Inactive |
| 96 | 6TOS | *OX1R* | B | Human | Inactive |
| 97 | 6TOD | *OX1R* | A | Human | Inactive |
| 98 | 6TQ4 | *OX1R* | B | Human | Inactive |
| 99 | 6TQ6 | *OX1R* | B | Human | Inactive |
| 100 | 6TO7 | *OX1R* | A | Human | Inactive |
| 101 | 6TQ9 | *OX1R* | B | Human | Inactive |
| 102 | 6TP4 | *OX1R* | B | Human | Inactive |
| 103 | 6TP3 | *OX1R* | A | Human | Inactive |
| 104 | 6TP6 | *OX1R* | B | Human | Inactive |
| 105 | 6TQ7 | *OX1R* | A | Human | Inactive |
| 106 | 6N4B | *CNR1* | R | Human | Active |
| 107 | 6KPG | *CNR1* | R | Human | Active |
| 108 | 6KPF | *CNR2* | R | Human | Active |
| 109 | 6PT0 | *CNR2* | R | Human | Active |
| 110 | 6LI2 | *GPR52* | A | Human | Intermediate |
| 111 | 6LI3 | *GPR52* | R | Human | Active |
| 112 | 5ZKB | *ACM2* | A | Human | Inactive |
| 113 | 5ZKC | *ACM2* | A | Human | Inactive |
| 114 | 5YC8 | *ACM2* | A | Human | Inactive |
| 115 | 5ZK3 | *ACM2* | A | Human | Inactive |
| 116 | 5ZK8 | *ACM2* | A | Human | Inactive |
| 117 | 6U1N | *ACM2* | R | Human | Active |
| 118 | 6OIK | *ACM2* | R | Human | Active |
| 119 | 4MQT | *ACM2* | A | Human | Active |
| 110 | 4MQS | *ACM2* | A | Human | Active |
| 120 | 6OIJ | *ACM1* | R | Human | Active |
| 121 | 4NC3 | *5HT2B* | A | Human | Intermediate |
| 122 | 5UIG | *AA2AR* | A | Human | Inactive |
| 123 | 3VG9 | *AA2AR* | A | Human | Inactive |
| 124 | 5IUB | *AA2AR* | A | Human | Inactive |
| 125 | 5IUA | *AA2AR* | A | Human | Inactive |
| 126 | 5OLZ | *AA2AR* | A | Human | Inactive |
| 127 | 5OLV | *AA2AR* | A | Human | Inactive |
| 128 | 5K2B | *AA2AR* | A | Human | Inactive |
| 129 | 6ZDV | *AA2AR* | A | Human | Inactive |
| 130 | 5OM4 | *AA2AR* | A | Human | Inactive |
| 131 | 5MZP | *AA2AR* | A | Human | Inactive |
| 132 | 5OLH | *AA2AR* | A | Human | Inactive |
| 133 | 5MZJ | *AA2AR* | A | Human | Inactive |
| 134 | 5OM1 | *AA2AR* | A | Human | Inactive |
| 135 | 5OLG | *AA2AR* | A | Human | Inactive |
| 136 | 6ZDR | *AA2AR* | A | Human | Inactive |
| 137 | 5NM2 | *AA2AR* | A | Human | Inactive |
| 138 | 6GT3 | *AA2AR* | A | Human | Inactive |
| 139 | 5JTB | *AA2AR* | A | Human | Inactive |
| 140 | 6PS7 | *AA2AR* | A | Human | Inactive |
| 141 | 5K2D | *AA2AR* | A | Human | Inactive |
| 142 | 5K2C | *AA2AR* | A | Human | Inactive |
| 143 | 6AQF | *AA2AR* | A | Human | Inactive |
| 144 | 5K2A | *AA2AR* | A | Human | Inactive |
| 145 | 6MH8 | *AA2AR* | A | Human | Inactive |
| 146 | 4EIY | *AA2AR* | A | Human | Inactive |
| 147 | 5VRA | *AA2AR* | A | Human | Inactive |
| 148 | 6JZH | *AA2AR* | A | Human | Inactive |
| 149 | 5UVI | *AA2AR* | A | Human | Inactive |
| 150 | 5OLO | *AA2AR* | A | Human | Inactive |
| 151 | 5NLX | *AA2AR* | A | Human | Inactive |
| 152 | 5NM4 | *AA2AR* | A | Human | Inactive |
| 153 | 3REY | *AA2AR* | A | Human | Inactive |
| 154 | 3PWH | *AA2AR* | A | Human | Inactive |
| 155 | 3UZC | *AA2AR* | A | Human | Inactive |
| 156 | 3RFM | *AA2AR* | A | Human | Inactive |
| 157 | 4UHR | *AA2AR* | A | Human | Intermediate |
| 158 | 2YDV | *AA2AR* | A | Human | Intermediate |
| 159 | 5UEN | *AA1R* | B | Human | Inactive |
| 160 | 6D9H | *AA1R* | R | Human | Active |

*Protein names corresponding to the listed genes are (using* [*https://www.genecards.org/*](https://www.genecards.org/)*): OPSD: rhodopsin; CLTR2: Cysteinyl Leukotriene Receptor 2; P2RY1: Purinergic Receptor P2Y ;* [*HTR2B*](https://www.genecards.org/cgi-bin/carddisp.pl?gene=HTR2B&keywords=5ht2b)*L: 5-Hydroxytryptamine (serotonin) Receptor 2B; SUCNR1: Succinate Receptor 1; US28: GPCR homologue US28; APJ: Apelin receptor; CCR2: CC Chemokine Receptor 2; CCR5: CC Chemokine Receptor 5; CCR6: CC Chemokine Receptor 6; CXCR2: CXC Chemokine Receptor 2; AGTR1: Angiotensin II Receptor Type 1; AGTR2: Angiotensin II Receptor Type 2; OPRX: Nociceptin receptor; OPRK: Kappa-type opioid receptor; OPRM: Mu-type opioid receptor; FPR2: N-formyl peptide receptor 2; C5AR1: C5a anaphylatoxin chemotactic receptor 1; NTR1: Neurotensin receptor type 1; OX1R: Orexin/Hypocretin receptor type 1; CNR1/2: Cannabinoid receptor 1/2; GPR52: G-protein coupled receptor 52; ACM2: Muscarinic acetylcholine receptor; 5HT2B: 5-hydroxytryptamine receptor 2B; AA2AR: Adenosine A_2A_ receptor; AA1R: Adenosine A_1_ receptor.*
