# Supplementary material for: Activation and Speciation Mechanisms in Class A GPCRs
Source: J Mol Biol. Author manuscript; Available in PMC 2023 Apr 25. (PMC10129049; doi:10.1016/j.jmb.2022.167690)
Supplement: Supplementary figures [file NIHMS1881903-supplement-Supplementary_figures.pdf]

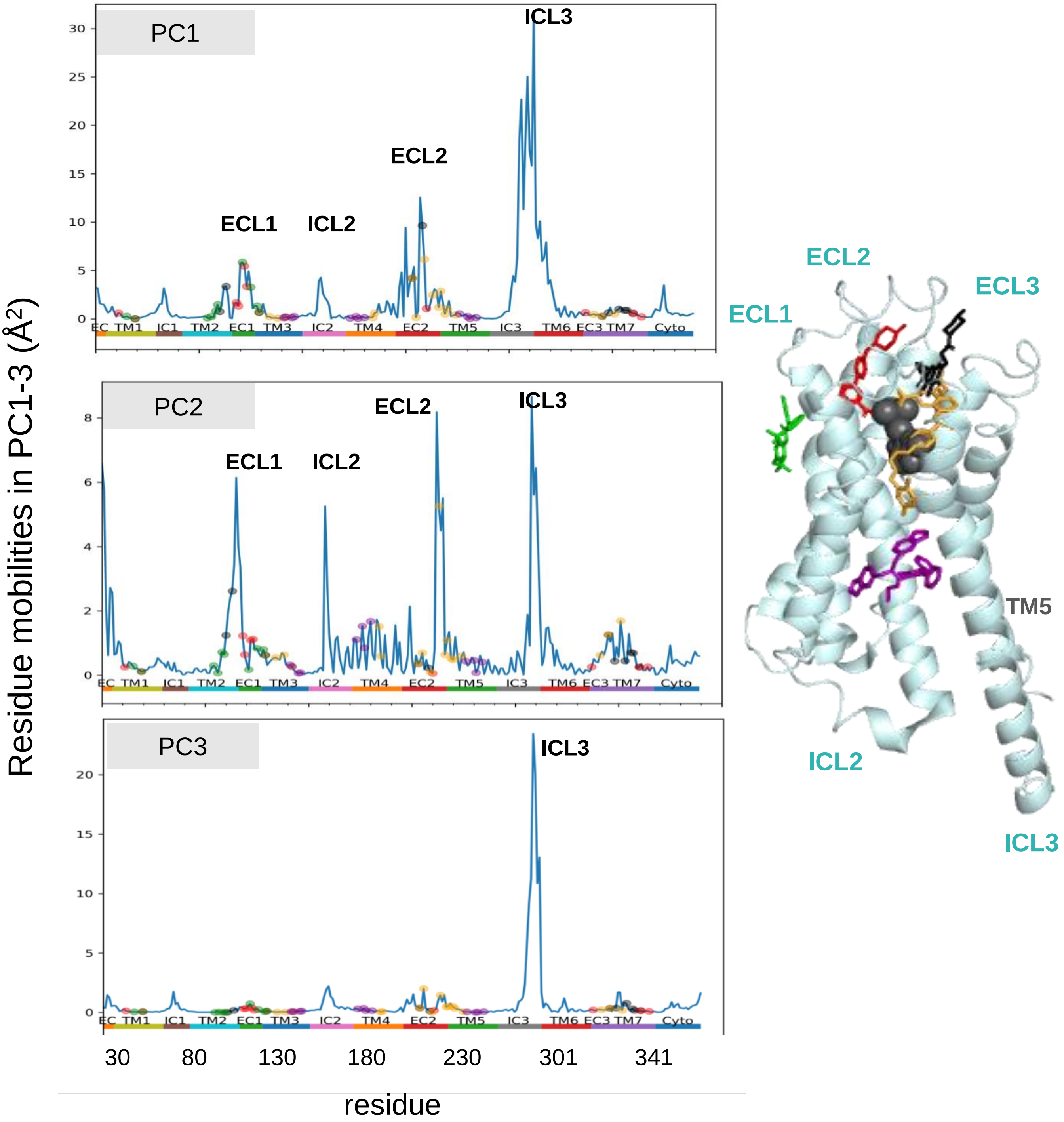


Supplementary Fig S1

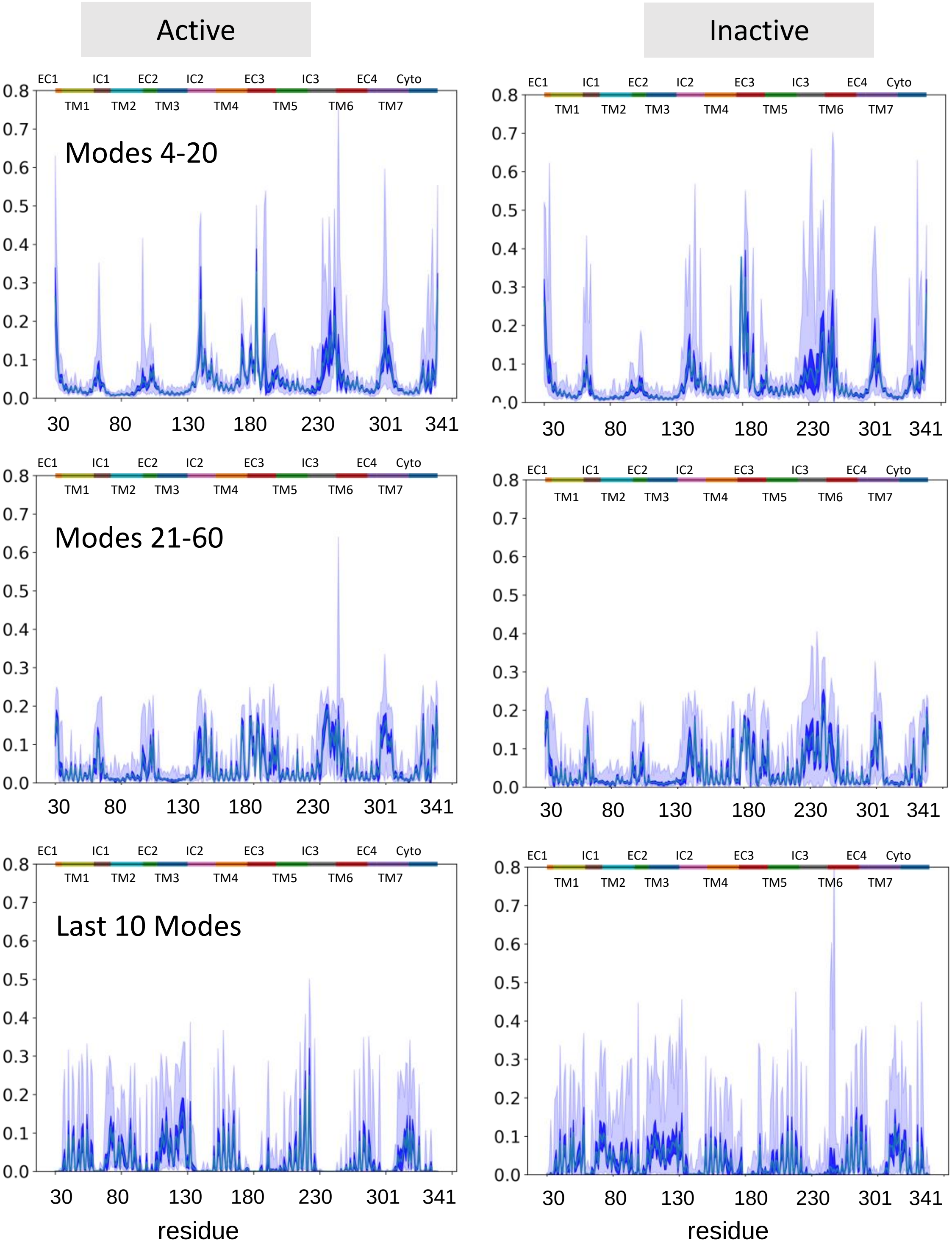


Supplementary Fig S2

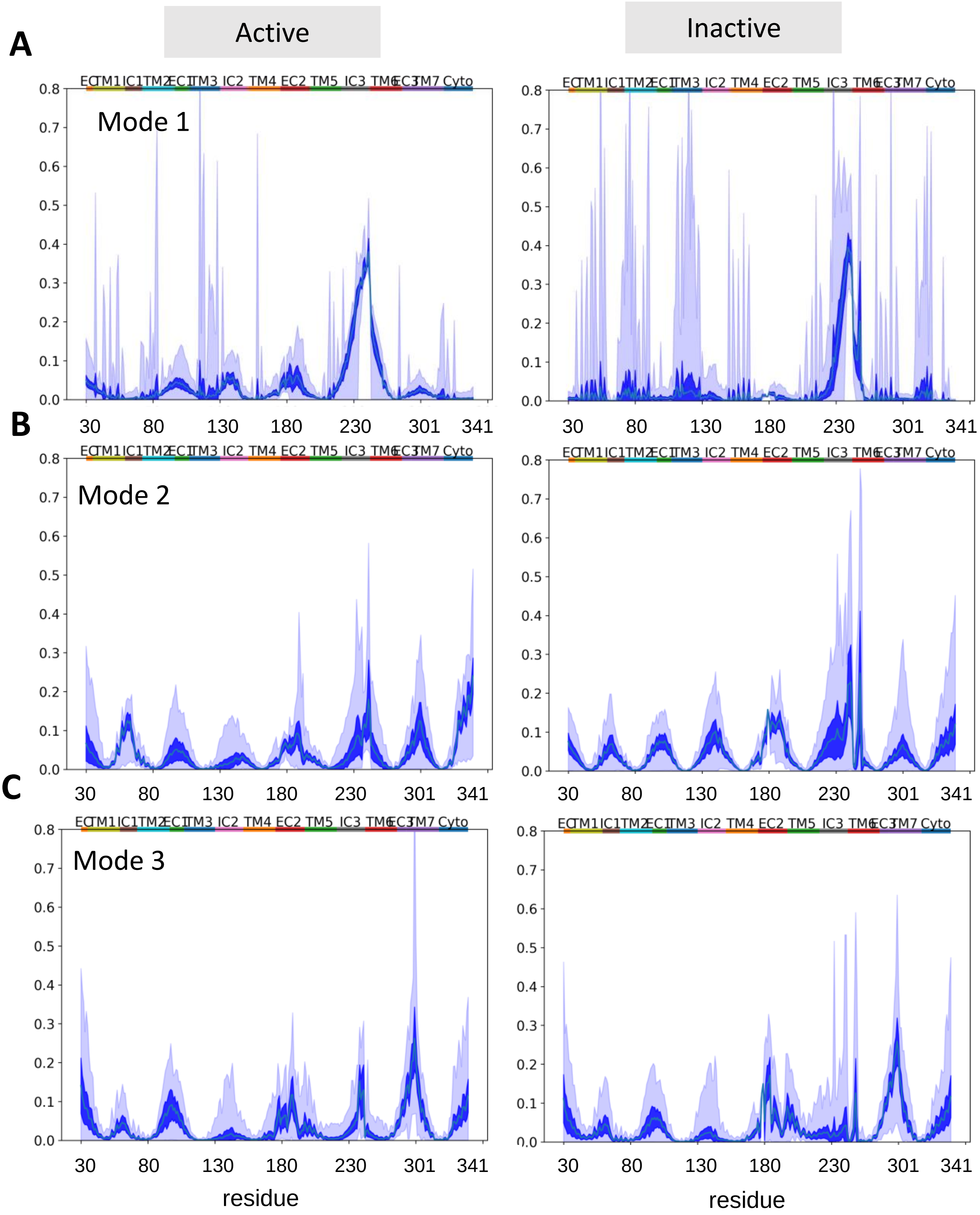


Supplementary Fig S3

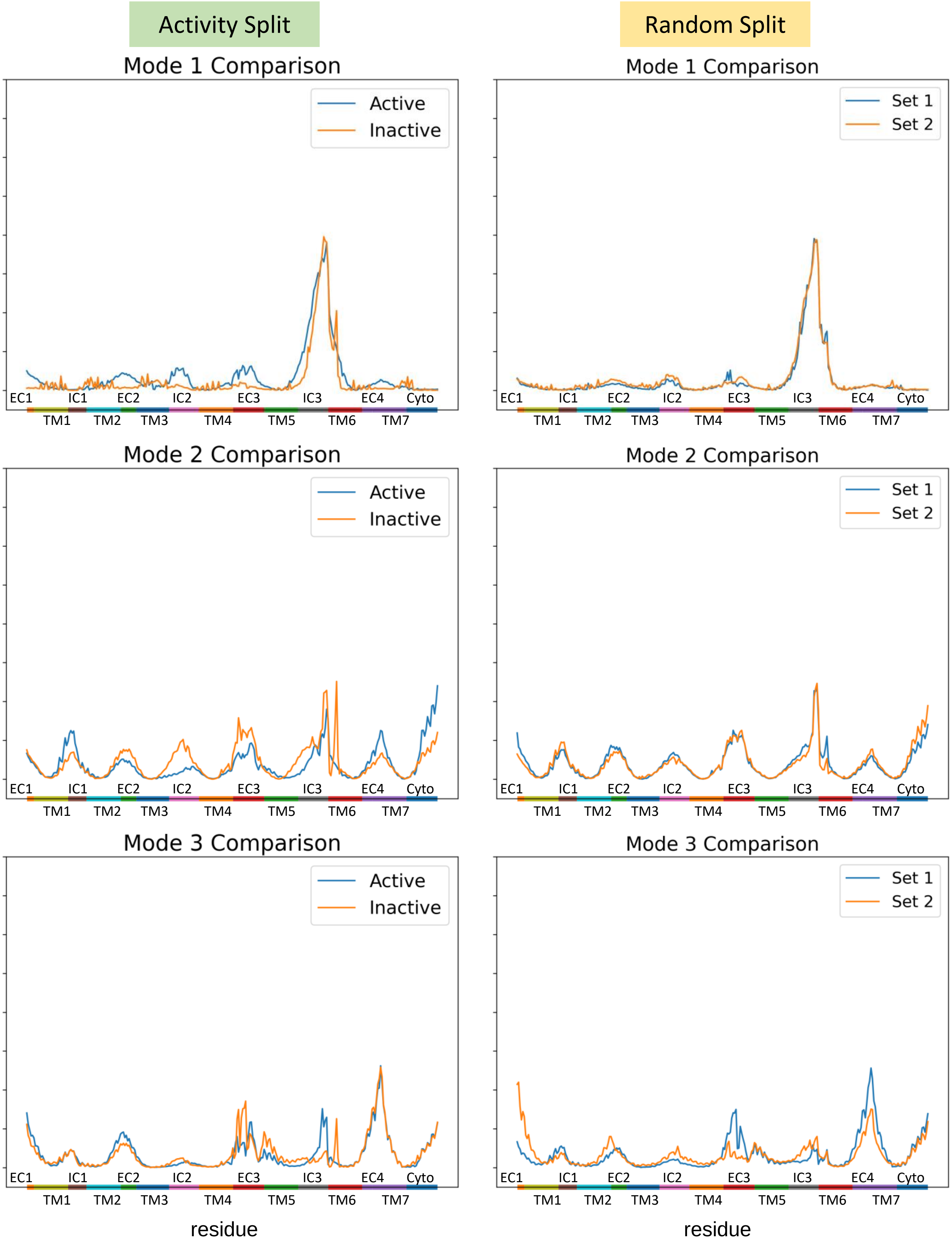


Supplementary Fig S4
