# Supplementary material for: Activation and Speciation Mechanisms in Class A GPCRs
Source: J Mol Biol. Author manuscript; Available in PMC 2023 Apr 25. (PMC10129049; doi:10.1016/j.jmb.2022.167690)
Supplement: Supplemental Movie 1 [file NIHMS1881903-supplement-Supplemental_Movie_1.pptx]

## Slide 1
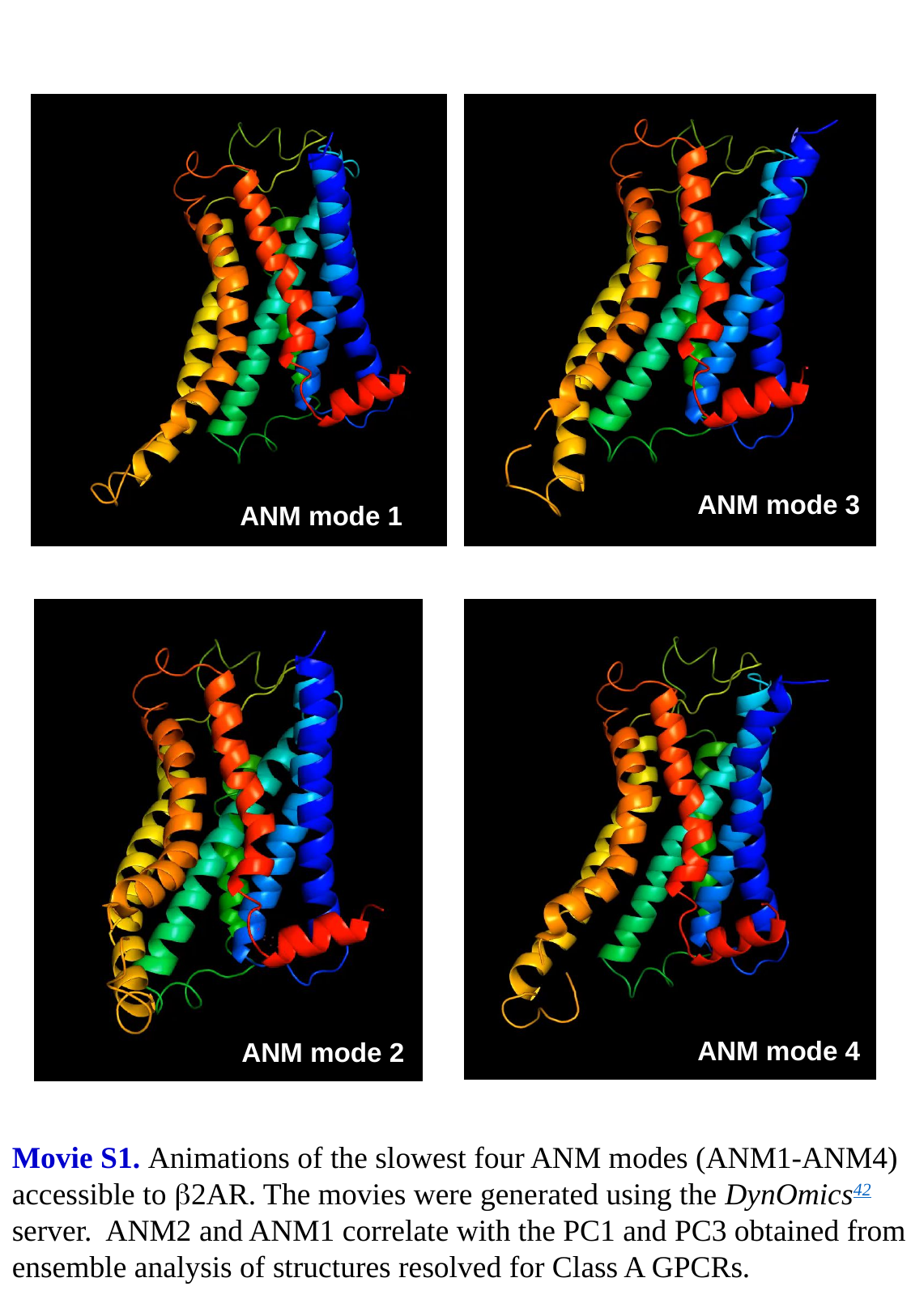

ANM mode 3
ANM mode 1
ANM mode 4
ANM mode 2
Movie S1. Animations of the slowest four ANM modes (ANM1-ANM4) accessible to b2AR. The movies were generated using the DynOmics42 server. ANM2 and ANM1 correlate with the PC1 and PC3 obtained from ensemble analysis of structures resolved for Class A GPCRs.
